# Intraoperative Hemi-Diaphragm Electrical Stimulation Demonstrates Attenuated Mitochondrial Function without Change in Oxidative Stress in Cardiothoracic Surgery Patients

**DOI:** 10.3390/antiox12051009

**Published:** 2023-04-27

**Authors:** Robert T. Mankowski, Stephanie E. Wohlgemuth, Guilherme Bresciani, A. Daniel Martin, George Arnaoutakis, Tomas Martin, Eric Jeng, Leonardo Ferreira, Tiago Machuca, Mindaugas Rackauskas, Ashley J. Smuder, Thomas Beaver, Christiaan Leeuwenburgh, Barbara K. Smith

**Affiliations:** 1 Department of Physiology and Aging, University of Florida, Gainesville, FL 32611, USA; 2 Department of Applied Physiology and Kinesiology, University of Florida, Gainesville, FL 32611, USA; 3 Department of Physical Therapy, University of Florida, Gainesville, FL 32611, USA; 4 Department of Surgery, University of Florida, Gainesville, FL 32611, USA

**Keywords:** respiration, mitophagy, VIDD, cardiothoracic surgery, biopsy

## Abstract

Mechanical ventilation during cardiothoracic surgery is life-saving but can lead to ventilator-induced diaphragm dysfunction (VIDD) and prolong ventilator weaning and hospital length of stay. Intraoperative phrenic nerve stimulation may preserve diaphragm force production to offset VIDD; we also investigated changes in mitochondrial function after stimulation. During cardiothoracic surgeries (*n* = 21), supramaximal, unilateral phrenic nerve stimulation was performed every 30 min for 1 min. Diaphragm biopsies were collected after the last stimulation and analyzed for mitochondrial respiration in permeabilized fibers and protein expression and enzymatic activity of biomarkers of oxidative stress and mitophagy. Patients received, on average, 6.2 ± 1.9 stimulation bouts. Stimulated hemidiaphragms showed lower leak respiration, maximum electron transport system (ETS) capacities, oxidative phosphorylation (OXPHOS), and spare capacity compared with unstimulated sides. There were no significant differences between mitochondrial enzyme activities and oxidative stress and mitophagy protein expression levels. Intraoperative phrenic nerve electrical stimulation led to an acute decrease of mitochondrial respiration in the stimulated hemidiaphragm, without differences in biomarkers of mitophagy or oxidative stress. Future studies warrant investigating optimal stimulation doses and testing post-operative chronic stimulation effects on weaning from the ventilator and rehabilitation outcomes.

## 1. Background

Among different types of cardiothoracic surgeries, coronary artery bypass grafting (CABG) surgery accounts for more than 50%, with approximately 400,000 procedures performed in the United States per year, as a result of coronary artery disease, most prevalent in older adults, 65 years and older [1]. An uncomplicated CABG surgery lasts approximately four hours, during which a patient is supported by mechanical ventilation, and the diaphragm muscle does not contract but moves passively with reduced blood flow, delivering oxygen and energy nutrients [2,3]. Mechanical-ventilation-acquired diaphragm weakness prolongs the hospital stay and rehabilitation and, thus, increases the burden on the healthcare system and takes a toll on the patient’s health and quality of life [2].

The emergence of diaphragm contractile dysfunction following mechanical ventilation is termed ventilator-induced diaphragm dysfunction (VIDD). The exact mechanisms of VIDD are unclear, but the body of evidence suggests that mitochondrial dysfunction, oxidative stress, and catabolic protein expression contribute to impaired diaphragmatic contractile function [4,5]. Dysregulated mitochondrial dynamics through impaired fission and fusion has been suggested as a cause of mitochondrial dysfunction and oxidative stress in the immobilized diaphragm during mechanical ventilation [6]. Others have shown that diaphragm weakness was caused by contractile dysfunction without mitochondrial dysfunction or oxidative stress [7].

There is no established clinical therapy to prevent diaphragm weakness during a surgery. Our group has previously demonstrated in a pilot study of older adults with respiratory comorbidities that electrically induced hemidiaphragm contractions during a cardiothoracic surgery improved or maintained mitochondrial respiration compared with the unstimulated side [8]. In addition, the stimulated side exhibited lower levels of oxidative stress and elevated expression of proteins related to autophagy, the process important for removing dysfunctional cell components, such as dysfunctional mitochondria, which are a source of oxidative stress [9]. These observations led to a controlled study of the effects of unilateral phrenic stimulation on mitochondrial respiration, mitophagy, mitochondrial enzymes, and markers of oxidative stress and protein catabolism. In contrast to the pilot study, most participants had normal pulmonary function tests or maximal inspiratory pressure. We hypothesized that the stimulated hemidiaphragm would increase levels of mitochondrial respiration and mitochondrial enzymes with concurrent reductions in mitophagy, oxidative damage, and protein catabolism markers.

## 2. Methods

### 2.1. Study Design

This was a controlled study (NCT 03303040) of intraoperative unilateral phrenic stimulation on diaphragm fiber remodeling, approved by the University of Florida Institutional Review Board. Adults between the ages of 18 and 80 years who were scheduled for cardiovascular or lung transplant surgery were eligible to participate. Exclusion criteria included a history of surgery to the diaphragm or pleura, New York Heart Association Class IV heart failure, chronic kidney disease with elevated serum creatinine (>1.6 mg/dL), body mass index (BMI) < 20 or >40 kg/m^2^, uncontrolled diabetes or thyroid disease, neuromuscular disease, current cancer treatment, or administration of immunosuppressants, corticosteroids, or aminoglycoside antibiotics in the 28 days preceding surgery. While severe obstructive airway disease (forced expiratory volume in the first second, FEV1 <40% of predicted) was exclusionary for cardiac surgery, this was not tested in transplant candidates. Each subject provided written informed consent prior to participation.

### 2.2. Screening Procedures

Consented subjects scheduled for cardiovascular surgery were screened preoperatively with seated forced vital capacity (FVC) and maximal inspiratory pressure (MIP) tests. Subjects repeated each test 3–5 times, and the highest effort was recorded. Subjects who could not achieve FEV1 >40% of predicted or MIP within the lower limit of normal age-predicted value [10,11] were ineligible to continue participation. Participants undergoing lung transplantation had previously undergone serial pulmonary function testing as part of clinical management of their disease surveillance and were not required to complete additional pre-operative screening.

### 2.3. Phrenic Stimulation

Subjects underwent unilateral 1 min phrenic stimulation during open cardiothoracic surgery, starting from the initial exposure of the phrenic nerves and continuing every 30 min until completion of the surgical repair. Intermittent twitch simulations of the right or left phrenic nerve were achieved using temporary pacing electrodes and an external cardiac pacemaker (Medtronic 5388, Minneapolis, MN, USA) set at pulse width: 1.5 ms, frequency: 30 pulses/minute, duration of stimulation: 1 min, and ventricular amplitude: twice the level of observable hemidiaphragm twitch contraction (maximum amplitude: 25 mA). The stimulated side was determined by the surgeon and based on the surgical procedures, other clinical equipment already in the operative suite, and phrenic nerve accessibility.

### 2.4. Diaphragm Biopsies

Diaphragm biopsies were obtained from participants who underwent four or more stimulation bouts. Full-thickness diaphragm biopsies were obtained from the stimulated and unstimulated portions of the costal diaphragm, approximately 30 min after the final phrenic stimulation bout. Specimens were placed immediately in chilled Buffer X (for composition see below, “Mitochondrial Respiration”), debrided of fat and connective tissue, divided, and weighed. Samples designated for mitochondrial respiration were stored in fresh Buffer X and immediately transported to the laboratory for further preparation for fresh tissue respirometry. Tissue reserved for protein, mitophagy, and oxidative stress studies were flash-frozen in liquid nitrogen and stored at −80 °C until analysis.

### 2.5. Mitochondrial Respiration

Tissue preparation: Samples were cleaned of adipose and connective tissue and immersed in ice-cold Buffer X (2.77 mM CaK_2_EGTA, 7.23 mM K_2_EGTA, 5.77 mM Na_2_ATP, 6.56 mM MgCl_2_·6H_2_O, 20 mM taurine, 15 mM Na_2_Phosphocreatine, 20 mM imidazole, 0.5 mM dithiothreitol, 50 mM K-MES, and 35 mM KCl; pH adjusted at 0 °C to 7.1 using 5 N KOH; 295 mosmol/kg H_2_O). They were inspected using a dissection microscope and further cleaned from adipose and connective tissue, if necessary. Small muscle fiber bundles were teased using a pair of fine forceps [12], transferred to ice-cold permeabilization solution (50 µg saponin/mL Buffer X), and incubated for 15 min at 4 °C on a rotator. Permeabilization was followed by a 10 min wash step at 4 °C on a rotator in mitochondrial respiration medium (Buffer Z; 110 mM K-MES, 35 mM KCl, 1 mM EGTA, 5 mM K_2_HPO_4_, 3 mM MgCl_2_·6H_2_O, 0.05 mM pyruvate, 0.02 mM malate with 0.5 mg mL, and 1 BSA (pH, 7.1; 295 mosmol/kg H_2_O)). Bundles were gently blotted dry using filter paper and weighed. Bundles (between 1 and 14 mg per respirometer chamber) were immediately transferred to previously calibrated oxygraph chambers containing Buffer Z and maintained at 37 °C (Oroboros O2K; Oroboros Instruments, Innsbruck, Austria).

Respirometry: Mitochondrial respiration was determined in duplicate, when possible (14 out of 20 subjects), under hyperoxic conditions (290–500 µM O_2_) as previously described [13]. Oxygen consumption rate (OCR; ρmol/s/mg tissue) was measured using the following substrate-uncoupler-inhibitor-titration (SUIT) protocol (concentration of reagents noted in parenthesis are final within chambers): (1) LEAK (L) respiration was assessed after tricarboxylic acid (TCA) cycle stimulation with NADH-linked substrates pyruvate (5 mM) and malate (2 mM) to support electron flow through Complex I (CI) of the electron transport system (ETS; E); (2) oxidative phosphorylation (OXPHOS; P) was stimulated with adenosine diphosphate (ADP; 2.5 mM) and recorded as PCI; (3) mitochondrial (mt) outer membrane integrity was tested by cytochrome c (10 µM) addition; (4) addition of succinate (10 mM) supported convergent electron flow through Complexes I and II of the ETS (OXPHOS; PCI + II); (5) the uncoupler carbonyl cyanide 4-(trifluoromethoxy) phenylhydrazone (FCCP; 0.5 µL of a 0.1 mM stock solution) was titrated step-wise until maximum uncoupled respiration was reached and recorded as maximum ETS capacity (ECI + II); (6) addition of rotenone (0.5 µM) inhibited Complex I of the ETS, and the remaining OCR was recorded as maximum ETS capacity supported by Complex II substrate (ECII; recorded from 12 and 11 samples of unstimulated and stimulated diaphragm samples, respectively); and (7) finally, addition of antimycin A (2.5 µM) inhibited Complex III of the ETS and, thereby, all electron transport to Complex IV; the remaining OCR was recorded as residual, non-mitochondrial oxygen consumption (ROX) and subtracted from all preceding respiratory states. Oxygen consumption data were acquired and analyzed using DatLab v.7.0 (Oroboros). Complex II (CII) OXPHOS capacity (PCII) was calculated as PCI + II − PCI. ATP-linked respiration with Complex I and Complex I + II substrates were calculated as (PCI − L; PCI/citrate synthase (CS) − L/CS) and (PCI + II − L; PCI + II/CS − L/CS); spare capacity as (ECI + II − PCI + II; ECI + II/CS − PCI + II/CS); and coupling efficiency as 1 − (L/PCI + II)**.**

### 2.6. Enzymatic Activity

For determination of the enzymatic activity of citrate synthase (CS), cytosolic (c), and mitochondrial (mt) aconitase, approx. 30 mg of muscle tissue was homogenized on ice with 10–15 passes in a Dounce glass homogenizer containing assay buffer (1:20 *w*/*v*) supplied by the assay kits’ manufacturer (Abcam, Cambridge, MA, USA; Citrate synthase #ab239712; aconitase #ab83459). The sample was centrifuged for 15 min at 20,000× *g* and 4 °C to clear the homogenate. The supernatant (c fraction) was transferred to a clean tube for c-aconitase activity measurement. The pellet was resuspended in assay buffer (1:10 *w*/*v*) and sonicated for 20 s (60 Sonic dismembrator, Fisher Scientific), followed by centrifugation for 5 min at 800× *g* and 4 °C. The supernatant (mt fraction) was transferred to a clean tube for mt-aconitase and CS activity measurements. Sample protein concentration was determined with the Bradford colorimetric assay, and samples were stored at −80 °C until enzyme activity measurement. Enzymatic activity was measured according to the manufacturer’s instructions and are expressed in mU/mg protein.

### 2.7. Determination of Selected Proteins by Immunodetection

Protein content of selected targets in muscle samples was measured by traditional Western blot (4-Hydroxynonenal (4-HNE)) as described previously [14], or by automated, capillary-based immunoassay (PINK-1, Parkin, or p62/SQSTM1 (p62)) using a Jess System (ProteinSimple, San Jose, CA, USA) and following the manufacturer’s instructions with slight modifications [15]. The Jess System utilizes cartridges containing 25 individual capillaries, in which all steps of the immunoassay occur, including size separation, immunodetection, and protein normalization. All assay steps were performed in a 400 nL volume within a single capillary. For both immunodetection methodologies, whole-tissue homogenates were prepared by extracting tissue protein in extraction buffer (20 mM HEPES, pH 7.4, 2 mM EGTA, 1% Triton X-100, 2% glycerol, 50 mM β-glycerophosphate, 1× Halt-protease, and phosphatase inhibitor cocktail (Thermo Scientific Cat#: 1861280)). Briefly, 20–30 mg frozen muscle tissue was quickly immersed in a pre-cooled vial filled with extraction buffer (1:20 *w*/*v*) and zirconium beads (Ø 3 mm) and placed in a BeadBug™ homogenizer (beads and homogenizer from Benchmark Scientific, Sayreville, NJ, USA). Tissue was homogenized five times at setting 4000 for 30 s each, with intermittent cooling for 1 min on ice. Subsequently, the homogenate was cleared by brief centrifugation, sonicated ten times for ~3 s at setting 4 (60 Sonic dismembrator), and centrifuged at 10,000× *g* for 10 min at 4 °C. Total protein content of the resulting supernatant was determined via Bradford colorimetric assay. Samples were diluted in Laemmli sample buffer (Bio-Rad, Hercules, CA, USA, #161-0747) supplemented with β-mercaptoethanol for traditional Western blot or sample buffer and Fluorescent Master Mix (ProteinSimple) for Jess, and proteins were denatured at 95 °C for 5 min. For traditional Western blot immunodetection, 50 µg protein was loaded onto a polyacrylamide gel (Bio-Rad), while sample protein concentrations of 0.25 and 0.75 µg/µL were loaded on the Jess separation module (12 to 230 kDa; ProteinSimple). The following commercially available primary antibodies were used: 4-HNE (R&D Systems, Minneapolis, MN, USA; #MAB3249; 1:1000), PINK-1 (Novus Biologicals, Littleton, CO, USA, #NB100-644SS; 1:50), Parkin (Abcam; #ab77924; 1:100), and p62 (MilliporeSigma, Burlington, MA, USA; #P0067; 1:600). HRP-conjugated secondary antibodies for traditional Western blot immunodetection were acquired from Cell Signaling (anti-rabbit IgG #7074, 1:5000 and anti-mouse IgG #7076, 1:5000) and acquired as ready-to-use reagents from ProteinSimple for the Jess system. All other reagents and supplies used for traditional Western blots were acquired from Bio-Rad (Hercules, CA, USA) and from ProteinSimple for the automated procedures on the Jess System. Each antibody was validated for optimal concentration using a calibration curve of serial homogenate dilutions to ensure linearity of signal quantification as well as optimal antibody dilution. Quantification of protein expression for traditional Western blots, using spot density of the target bands, was performed with Image Lab 6.0 software (Bio-Rad Laboratories) and normalized to the amount of protein loaded in each lane, as determined by densitometric analysis of the corresponding Ponceau S-stained membranes [16,17]. Quantification of the resulting electropherograms for the Jess System was performed with Compass for SW software (v4.1.0; ProteinSimple) using a Gaussian peak fit distribution for area under the curve. The peak area for each capillary was normalized to total protein using the system’s Protein Normalization capability and reagents for the Jess System.

### 2.8. Statistical Analysis

The distribution of the data was assessed with the Shapiro–Wilk test. Differences in normally distributed data were tested with paired t-tests, and Wilcoxon tests were used for non-normal distributions. Data are expressed as mean (standard deviation and standard error of the mean), statistical significance was *p* < 0.05, and we define a “trend” (as 0.05 < *p* < 0.1).

## 3. Results

### 3.1. Patient Characteristics and Tissue Acquisition

Twenty-one patients (nine females, twelve males, mean age: 59 ± 11 years) consented to participate, completed intraoperative stimulation, and provided diaphragm biopsies (Table 1). Table 2 details parameters of the intraoperative stimulation. Subjects received an average 6.2 ± 1.9 stimulation bouts at a current intensity of 18.0 ± 5.4 mA. The average time between intubation and muscle biopsy acquisition was 278 ± 65 min.

### 3.2. Mitochondrial Respiration

**Figure 1:**
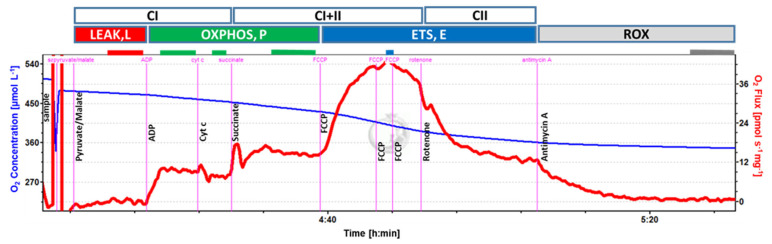
**Respirometry protocol with permeabilized fibers from diaphragm muscle.** Typical trace of oxygen consumption after permeabilized fiber preparation with pyruvate/malate and succinate substrate combinations to support electron flow through Complex I (CI) and Complex I + II (CI + II), respectively, of the mitochondrial electron transport system (ETS) and its activation by ADP. Cytochrome *c* was added as a quality control (see text for details); FCCP to induce uncoupling and evaluate ETS capacity with CI + II substrates and CII substrate (after inhibition of CI with Rotenone), subsequentially; and Antimycin A (inhibitor of complex III of the ETS) to evaluate residual oxygen consumption (ROX). The blue line represents the oxygen concentration (μmol L^−1^), and the red line represents the muscle-mass-specific O_2_ flux (pmol O_2_ s^−1^ mg^−1^; negative slope of the blue line normalized to tissue weight). Marked sections (solid lines above trace) correspond to steady-state fluxes at different coupling states (L, P, and E; see text for explanations).

Integrative mitochondrial respiration (see Figure 1) (oxygen consumption per mg tissue wet weight) during OXPHOS with Complex I and with Complex II substrates, respectively (PCI and PCII), was not different between stimulated and unstimulated sides. Leak respiration (L; *p* = 0.042) and maximum ETS capacities (ECI + II, *p* = 0.039; ECII, *p* = 0.043) were lower, and OXPHOS with combined Complex I and II substrates (PCI + II) trended to be lower (*p* = 0.065) in the stimulated compared with the unstimulated diaphragm side (Table 3). Similarly, when normalized to mitochondrial unit (intrinsic mitochondrial respiration using CS activity as a proxy measure for mitochondrial content [18]), L and PCI were significantly lower on the stimulated side (*p* = 0.027 and 0.042, respectively), and ECI + II and ECII tended to be lower (*p* = 0.083 for both; Table 3). Spare capacity, the increase of maximum ETS capacity above maximum OXPHOS, tended to be lower on the stimulated side (−7.5% and −11%, *p* = 0.06 and 0.091, for integrative and intrinsic respiration, respectively; Table 3). Flux control ratios, oxygen flux normalized to maximum ETS capacity, coupling efficiency (1-L/P_CI + II_), as well as integrative and intrinsic ATP-linked respiration were not different between stimulated and unstimulated sides. Finally, CS activity per mg tissue (Table 3) and per mg protein did not differ between the stimulated and unstimulated diaphragm sides [18].

### 3.3. Oxidative Stress

**Figure 2:**
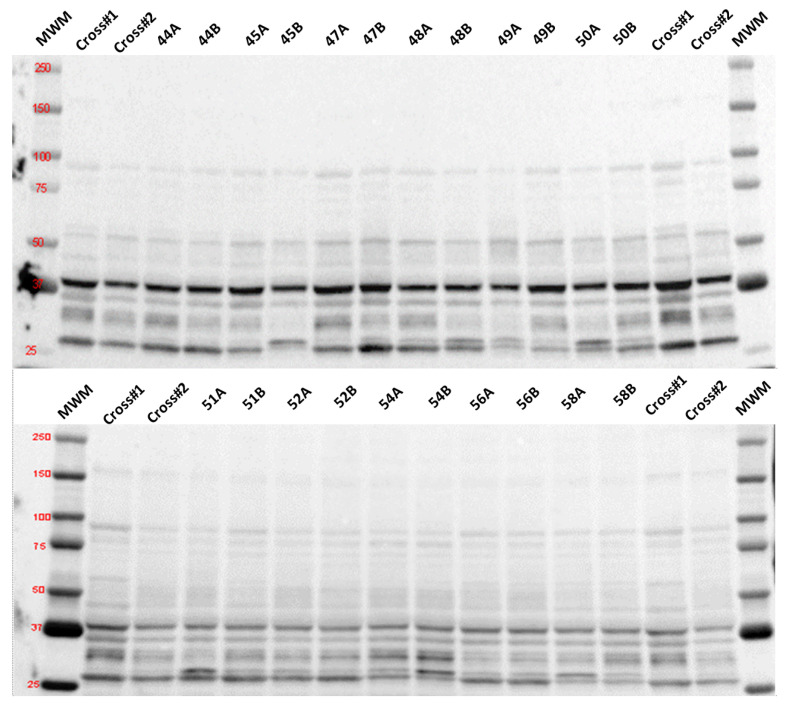
Immunoblots of 4-HNE-modified proteins in tissue lysate from muscle biopsies of stimulated and unstimulated side of the diaphragm. Numbers on top represent participant ID, with different letters (A,B) indicating stimulated and unstimulated side, respectively. Cross: cross samples that were run on both gels alongside the samples to allow comparison between the two immunoblots. MWM: molecular weight marker (numbers indicate molecular weight (kDa) of respective band). Blue box indicates area for densitometry (only one band shown as an example).

Enzymatic activity of mitochondrial aconitase was evaluated in twenty and eighteen subjects, respectively (unstimulated and stimulated sides). We determined a trend toward an attenuation of enzyme activity in the stimulated compared to the unstimulated side (−11%; *p* = 0.06; Table 3). Expression of 4-HNE (4-hydroxynonenal), as a marker of lipid peroxidation, was measured in eleven subjects and did not differ between groups (*p* = 0.41;) (see Figure 2).

### 3.4. Mitophagy

None of the mitophagy markers (p62, full-length PINK-1, cleaved PINK-1, or Parkin) evaluated in the eleven subjects were expressed differently between stimulated and unstimulated sides (p62: *p* = 0.683; PINK-1: *p* = 0.504; and Parkin: *p* = 0.967).

When comparing associations between expression of mitophagy markers and oxygen flux, we found that expression of p62 and full-length PINK-1 correlated with maximal ETS capacity in the stimulated side (ECI + II; p62: Pearson’s correlation coefficient r = −0.75, *p* = 0.033; full-length PINK-1: r = 0.69, *p* = 0.042), but there were no correlations in the unstimulated side. Maximal OXPHOS capacity (PCI + II) trended to correlate with expression of full-length PINK-1 (r = 0.65, *p* = 0.059) in the stimulated side and with cleaved PINK-1 (r = −0.63, *p* = 0.071) on the unstimulated side.

## 4. Discussion

The primary finding of this study is that electrical stimulation of the hemidiaphragm attenuated integrative and intrinsic mitochondrial respiratory function in the diaphragm muscle. Specifically, leak respiration and OXPHOS and ETS capacities were lower, and spare capacity was reduced after electrical stimulation in muscle biopsy samples from the stimulated side.

The current findings contrast with our previous work, where hourly hemidiaphragm stimulation over the course of prolonged cardiovascular surgery significantly increased State 3 (PCI) and State 4 (leak) respiration [8]. In the same subjects presented here, this supramaximal phrenic stimulation regimen offset contractile dysfunction and atrophy of slow diaphragm fibers [19]. A precise intraoperative quantification of the stimulation intensity is not feasible, but supramaximal stimulation was targeted to preserve maximal force production. While supramaximal twitch stimulations do not directly mimic spontaneous respiratory efforts, they were selected to reduce variability with a spontaneous or evoked submaximal contraction. Although we are not aware of existing data on mitochondrial capacity after hemidiaphragm electrical stimulation, there is evidence that other types of intense/maximal exercise bouts could acutely disrupt mitochondrial function in skeletal muscle. For example, acute high-intensity exercise (ergometer 5 km time trial) in young healthy subjects diminished ATP-linked respiratory capacity and spare capacity immediately after an exercise bout, without differences in CS activity or proton leak [20].

We then investigated whether the observed changes in oxidative phosphorylation were associated with mitochondrial stress and general cellular oxidative damage. Expression of 4-HNE, a marker of lipid peroxidation, was unaffected by electrical stimulation. Mitochondrial aconitase, which has been suggested to be a sensitive indicator of mitochondrial oxidative stress [21], displayed a lower enzymatic activity in samples from the stimulated side. Interestingly, there was a positive correlation of (full-length) PINK-1 with P_CI+II_ and E_CI+II_ in samples from the stimulated side, which was absent in those from the unstimulated side. Subcellular localization of full-length PINK-1 to the mitochondria is regulated by the mitochondrial membrane potential and increases with mitochondrial depolarization [22]. Once stabilized at the mitochondrial membrane, PINK-1 recruits Parkin to the damaged mitochondria, and the process of mitophagy, the removal of damaged mitochondria through autophagy, ensues [23]. The association and correlation of PINK-1 accumulation with higher respiratory activity that we observed only in electrically stimulated muscle fibers, together with less functional mitochondrial aconitase, could be indicative of increased oxidative stress in the stimulated muscle fibers. We did not, however, detect an increase in Parkin or p62 protein, the latter a selective cargo receptor associated with autophagy and mitophagy [24], both being downstream of PINK-1 recruitment to the outer mitochondrial membrane. It is possible that the observed PINK-1 accumulation indicates an early stage of mitochondrial stress. In our previous work, we did not investigate markers of mitophagy but found an increase in autophagy markers Beclin-1 and LC3II/I ratio, suggesting induction of autophagy following the stimulation protocol [9]. While autophagy per se could be consistent with cellular damage, it could also be beneficial to skeletal muscle contractile function by facilitating removal of cellular materials damaged during stimulation or exercise [25].

While we did not find a similar increase in mitochondrial function in the current study, some key differences between the current study and our pilot project may have contributed to the discrepancy. These include differences in the volume/dose of stimulation, the severity of intraoperative cooling, and the baseline respiratory function of participants. Across a similar intraoperative time, an average of 3.4 ± 0.6 hourly stimulations were administered in the previous study, as compared with 6.2 ± 1.9 stimulations every 30 min in the current study. Despite similar stimulation intensities (17 ± 4 vs. 18 ± 5 mA) in the two studies, the number of stimulation bouts was >80% greater in the current study. Since the “dose” of stimulation necessary to optimize mitochondrial respiration has not yet been determined, we cannot verify whether the increased stimulation volume was detrimental to mitochondrial function.

In addition to the actual stimulation protocol, intraoperative temperature was another difference between the previous and current study that could explain the different outcomes. Although diaphragm biopsies were acquired after rewarming, the surgical procedures in the previous study were associated with more prolonged and extensive intraoperative cooling (31.3 °C ± 2.6 degrees versus 34.8 °C ± 1.1 degrees in the current trial). In animal studies, heating skeletal muscle by 5 °C decreased mitochondrial respiration in response to a fatty acid substrate without changes in CS enzyme activity [26]. In another study in isolated resting rat skeletal muscle, increasing temperature increased mitochondrial oxidative capacity and proton leak, which could explain decreased efficiency of OXPHOS [27].

Additionally, we speculate that differences in the baseline pulmonary function of the current study participants could contribute to the study findings. Despite similarities in age and BMI of subjects, average spirometry and maximal inspiratory pressures (a clinical estimate of strength) were normal in the current sample (FVC: 87 ± 14% predicted and maximal inspiratory pressure: 91 ± 23 cm H_2_O) and considerably higher than our prior reports (Ahn et al.: FVC: 54 ± 19% predicted, MIP: 62 ± 3 cm H_2_O; Mankowski et al.: FVC: 78 ± 17% predicted, MIP: 72 ± 15 cm H_2_O) [9,28]. Animal [29] and human [30] studies reveal a reduced skeletal muscle mitochondrial density, impaired mitochondrial respiration, and excessive oxidative stress in obstructive lung disease. Thus, the contraction stimulus may have elicited relatively smaller gains in muscle fiber function when starting from normal baseline diaphragm function. Since acute, intense exercise can transiently decrease mitochondrial function in skeletal muscle of both control and lung disease subjects [31], the observed differences between the studies are likely more complex, reflecting combined influences of both pre-existing patient function and the phrenic stimulation protocol.

While intraoperative phrenic stimulation led to only modest differences in mitochondrial function compared with the non-stimulated hemidiaphragm, mitochondrial respiration does not appear to significantly contribute to diaphragm contractile dysfunction in critically ill adults with prolonged mechanical ventilation [7]. However, intraoperative mechanical ventilation does not represent a pure model of VIDD, since other aspects of the surgical environment, including cardiopulmonary bypass, anesthesia, and hypothermia [32,33,34], may independently affect mitochondrial respiration, and the surgical process itself can alter the mechanics of the respiratory muscles [35].

### Strengths and Limitations

A strength of the study design is that it incorporates a clinically relevant model of mechanical ventilation that can lead to prolonged ventilatory support. One limitation of this research model is some necessary variability of the surgical environment required to meet each subject’s clinical needs. As a result, we note differences in the total amount of anesthetic, minimal core body temperature, and duration of the surgical procedure, which also impacted the total number of stimulations. Considerable heterogeneity was also observed in the study dependent metrics. Due to a limited sample size, we did not analyze our results to compare the effect of sex, which will be a plan for future larger studies. We evaluated the strength of association between surgical factors and subsequent mitochondrial respiration and protein expression but did not identify any significant associations that could account for the variable subject responses. Similarly, no subject characteristics could account for the observed variability in mitochondrial respiration, protein expression, mitophagy, or enzyme activity. Due to the limited amount of diaphragm muscle tissue, we were not able to analyze it for more oxidative stress biomarkers or mitochondrial enzymes.

While most subjects were recruited in advance of open cardiothoracic surgery, two participants in the study (Subjects 8 and 9) underwent bilateral lung transplantation due to end-stage restrictive pulmonary disease. The remaining study participants were relatively young and active, with few pulmonary comorbidities and, thus, considered to have a lower risk for post-operative ventilatory failure. We compared the study-dependent measures of the full sample with those of the transplant-patient-excluded sample and found that excluding these subjects did not change the primary study findings.

Unilateral phrenic stimulation reduced variability by using each subject’s non-stimulated hemidiaphragm as an inactive control. Supramaximal stimulation of one hemidiaphragm could induce passive stretch of the unstimulated side via forced transduction from the central tendon [36]. Indeed, intermittent passive stretch elicited by unilateral diaphragm denervation elicits high passive stretch and titin-mediated fiber hypertrophy (both cross-sectional and longitudinal) [37]. Passive stretch also increases mitochondrial calcium concentrations, which stimulates mitochondrial respiration [38]. Thus, stimulation-induced compensatory changes in mitochondrial respiration, mitophagy, and protein expression may have been underestimated. Additionally, we realize that the tested supramaximal twitch diaphragm stimulation does not emulate physiological diaphragm contractions during breathing. The goal of this intervention was to investigate whether contractile activity of the diaphragm during mechanical ventilation induces biological changes that may preserve diaphragm function. Further studies of different stimulation protocols (e.g., frequency and intensity of stimulation or bilateral phrenic stimulation) may reveal more clinically relevant benefits.

## 5. Conclusions

In conclusion, this unique clinical trial demonstrated that the intraoperative phrenic nerve electrical stimulation led to an acute decrease of mitochondrial respiration in the stimulated hemidiaphragm, without differences in biomarkers of mitophagy and oxidative stress. Future studies are warranted to personalize the dose of intraoperative stimulations and study the impact of diaphragm electrical stimulation training after surgery to improve weaning from the ventilator and shorten the ICU stay.

## Figures and Tables

**Table 1:** Demographic data and respiratory function parameters of patients.

Patient #	Age (Yrs)/Sex	BMI (Kg/m^2^)	%FVC	%FEV1	PI Max (cm H_2_O)	Intubation to Biopsy (Min)	Surgery Type
1	61/F	36.2	105	96	83	450	Maze, MVR, AVR
2	60/M	29.6	85	90	85	289	Ascending aorta graft, aortic root replacement, AVR
3	71/F	28.8	78	86	67	249	CABG × 3
4	76/F	29.6	79	83	79	232	CABG × 3
5	74/F	21.4	98	87	83	319	CABG × 1, MVR
6	62/F	30.8	100	101	72	295	CABG × 4
7	58/F	34.6	73	71	61	293	CABG × 3
8	62/M	28.9	-	-	-	281	Bilateral lung transplant
9	55/M	27.1	-	-	-	273	Bilateral lung transplant
10	69/M	27.5	65	58	92	240	AVR, Maze
11	47/M	32	101	97	120	203	AVR
12	55/M	27.9	78	64	76	230	Ascending aorta graft, aortic root replacement, AVR
13	35/M	34.5	90	89	155	358	CABG × 3
14	72/M	30.1	87	94	86	261	CABG × 2
15	56/F	31.8	81	79	83	233	Mitral repair
16	35/F	32.9	95	96	64	235	MVR
17	51/M	31.9	111	106	110	415	AVR, CABG × 3
18	49/M	22.6	78	68	93	269	Ascending aorta graft, aortic root, AVR, MVR, Maze
19	71/M	24.3	83	78	124	195	AVR, MVR
20	57/F	35.4	91	83	90	227	AVR, ascending aorta graft
21	63/M	28.2	92	94	80	286	CABG × 4

F: female; M: male; BMI: body mass index; %FVC: forced vital capacity; %FEV1: forced expiratory volume; PI Max: maximal inspiratory pressure; CABG: coronary artery bypass grafting; AVR: aortic valve replacement; MVR: mitral valve replacement.

**Table 2:** Intraoperative and stimulation characteristics of the sample.

Patient #	Intubation to First Stim (Min)	Cardio-Pulmonary Bypass (Min)	Average Stimulation (mA)	Number of Stims	Minimum Core Temp (C)	Core Temp at Biopsy (C)	Intubation to Biopsy (Min)
1	104	341	6.8	12	30.3	37.4	450
2	112	152	10.0	6	33.5	37.0	289
3	71	91	12.0	6	33.2	36.9	249
4	71	72	10.0	6	36.2	-	232
5	68	204	17.3	8	36.8	35.3	319
6	102	128	19.0	7	34.9	36.9	295
7	77	115	19.3	7	34.2	-	293
8	83	-	16.0	7	36.5	-	281
9	140	-	23.0	5	36.9	36.9	273
10	57	173	12.0	6	36.8	35.0	240
11	91	90	20.0	4	33.1	35.0	203
12	73	125	20.2	5	32.3	35.1	230
13	108	135	25.0	9	34.2	37.2	358
14	112	66	25.0	5	34.5	36.5	261
15	111	118	20.8	5	32.0	37.2	233
16	76	102	13.2	5	31.3	37.3	235
17	210	202	23.3	7	28.3	35.4	415
18	94	165	20.0	6	32.2	35.7	269
19	93	64	25.0	4	33.9	36.0	195
20	111	108	20.2	4	32.5	36.0	227
21	83	149	20.7	6	29.8	36.9	286
mean ± SD	98 ± 33	137 ± 65	18.0 ± 5.4	6.2 ± 1.9	33.5 ± 2.4	36.5 ± 0.8	278 ± 65

**Table 3:** Mitochondrial respiratory function, mitochondrial citrate synthase (CS), and aconitase activities in biopsy samples from unstimulated and stimulated diaphragm.

Corrected	Unstimulated Side (*n*)	Stimulated Side (*n*)	*p*-Value
**Flux (pmol/s/mg wwt)**			
L	3.5 ± 0.7 (17)	2.9 ± 0.6 (16)	0.135
PCI	9.6 ± 1.0 (19)	9.1 ± 0.7 (19)	0.155
PCII	8. 7 ± 0.9 (19)	8.1 ± 0.9 (19)	0.256
PCI + II	18.3 ± 1.5 (19)	17.2 ± 1.4 (19)	**0.065**
ECI + II	31.1 ± 3.5 (19)	27.1 ± 2.1 (19)	**0.026**
ECII	17.7 ± 2.2 (11)	14.2 ± 1.5 (12)	**0.032**
**Flux/unit CS activity (pmol/s/CS activity)**			
L	46.3 ± 10.2 (14)	35.9 ± 7.9 (15)	**0.052**
PCI	123.4 ± 12.6 (15)	110.3 ± 8.7 (18)	**0.042**
PCII	113.1 ± 15.3 (15)	100.4 ± 9.0 (18)	0.504
PCI + II	236.5 ± 21.5 (15)	210.7 ± 13.8 (18)	0.192
ECI + II	415.6 ± 61.5 (15)	339.3 ± 29.8 (18)	**0.083**
ECII	231.1 ± 41.7 (9)	179.0 ± 24.1 (11)	**0.083**
**Flux Control Ratio (Flux relative to ECI + II)**			
L	0.141 ± 0.038 (17)	0.122 ± 0.035 (16)	0.246
PCI	0.336 ± 0.030 (19)	0.351 ± 0.026 (19)	0.389
PCII	0.296 ± 0.029 (19)	0.308 ± 0.029 (19)	0.420
PCI + II	0.630 ± 0.032 (19)	0.659 ± 0.036 (19)	0.134
ECII	0.493 ± 0.051 (11)	0.493 ± 0.046 (12)	0.746
**ATP-linked Respiration (PCI)**	6.7 ± 1.1 (17)	7.2 ± 0.9 (15)	0.794
**ATP-linked Respiration (PCI + II)**	15.4 ± 1.8 (17)	14.2 ± 1.5 (16)	0.609
**ATP-linked Respiration (PCI/CS)**	82.2 ± 15.4 (14)	80.3 ± 12.5 (15)	0.537
**ATP-linked Respiration (PCI + II/CS)**	197.0 ± 25.9 (14)	174.1 ± 17.7 (15)	0.530
**Spare capacity (ECI + II−PCI + II)**	12.8 ± 2.5 (19)	9.9 ± 1.6 (19)	**0.039**
**Spare capacity (ECI + II/CS−PCI + II/CS)**	179.0 ± 44.5 (15)	128.6 ± 22.9 (18)	**0.048**
**Coupling efficiency (1-L/PCI + II)**	0.792 ± 0.05 (17)	0.817 ± 0.05 (16)	0.241
**CS activity/mg tissue (mU/mg wwt)**	0.084 ± 0.006 (18)	0.082 ± 0.004 (20)	0.626
**Mt-aconitase activity (mU/mg protein)**	0.196 ± 0.01 (18)	0.175 ± 0.01 (20)	**0.060**

Mitochondrial respiratory function is reported as oxygen consumption (flux) per tissue weight (pmol/s/mg wwt) and normalized to mitochondrial content (by proxy of citrate synthase activity; pmol/s/CS activity). Flux control ratios are calculated as flux relative to maximal electron transport capacity (ECI + II). ATP-linked respiration (per tissue weight and per mitochondrial unit, respectively) is calculated as leak-subtracted OXPHOS capacity with Complex I and Complex I + II supporting substrates. Spare capacity (per tissue weight and per mitochondrial unit) is calculated as maximal electron transport capacity (ECI + II) minus maximal OXPHOS capacity (PCI + II). Enzyme activities are reported as mU/mg tissue wwt (CS) or mU/mg protein (aconitase). Data are reported as mean ± SEM, and *p*-values result from a paired-t-test or a Wilcoxon matched-pairs signed rank test when samples were not normally distributed; differences with *p* ≤ 0.1 were considered a trend, and *p* ≤ 0.05 was considered significant. Significant and trending differences are bolded.

## Data Availability

The datasets used and/or analyzed during the current study are available from the corresponding author upon reasonable request.

## Abbreviations

VIDD: ventilator-induced diaphragm dysfunction; CABG: coronary artery bypass grafting; FEV1: forced expiratory volume in the first second; BMI: body mass index: FVC: forced vital capacity; MIP: maximal inspiratory pressure; FEV: forced expiratory volume; EGTA: egtazic acid; ATP: adenosine triphosphate; BSA: bovine serum albumin; OCR: oxygen consumption rate; SUIT: substrate-uncoupler-inhibitor-titration; TCA: tricarboxylic acid; NADH: reduced nicotinamide adenine dinucleotide; CI: Complex I; ETS: electron transport system; OXPHOS: oxidative phosphorylation; ADP: adenosine diphosphate; Mt: mitochondrial; ECI + II: maximum ETS capacity; ECII: maximum ETS capacity supported by Complex II substrate; ROX: non-mitochondrial oxygen consumption; CII: Complex II; CS: citrate synthase; 4-HNE: 4-Hydroxynonenal; LC3: microtubule-associated protein 1A/1B-light chain 3.
